# Genome Analysis of Haplotype D of *Candidatus* Liberibacter Solanacearum

**DOI:** 10.3389/fmicb.2018.02933

**Published:** 2018-12-10

**Authors:** Leron Katsir, Ruan Zhepu, Diego Santos Garcia, Alon Piasezky, Jiandong Jiang, Noa Sela, Shiri Freilich, Ofir Bahar

**Affiliations:** ^1^Department of Plant Pathology and Weed Research, Agricultural Research Organization, Volcani Center, Rishon LeZion, Israel; ^2^Newe Ya’ar Research Center, Agricultural Research Organization, Ramat Yishay, Israel; ^3^Department of Microbiology, College of Life Sciences, Nanjing Agricultural University, Nanjing, China; ^4^Department of Entomology, The Robert H. Smith Faculty of Agriculture, Food and Environment, The Hebrew University of Jerusalem, Rehovot, Israel; ^5^Department of Plant Pathology and Microbiology, The Robert H. Smith Faculty of Agriculture, Food and Environment, The Hebrew University of Jerusalem, Rehovot, Israel

**Keywords:** genomics, fastidious bacteria, plant pathogen, Liberibacter, psyllid

## Abstract

*Candidatus* Liberibacter solanacearum (Lso) haplotype D (LsoD) is a suspected bacterial pathogen, spread by the phloem-feeding psyllid *Bactericera trigonica* Hodkinson and found to infect carrot plants throughout the Mediterranean. Haplotype D is one of six haplotypes of Lso that each have specific and overlapping host preferences, disease symptoms, and psyllid vectors. Genotyping of rRNA genes has allowed for tracking the haplotype diversity of Lso and genome sequencing of several haplotypes has been performed to advance a comprehensive understanding of Lso diseases and of the phylogenetic relationships among the haplotypes. To further pursue that aim we have sequenced the genome of LsoD from its psyllid vector and report here its draft genome. Genome-based single nucleotide polymorphism analysis indicates LsoD is most closely related to the A haplotype. Genomic features and the metabolic potential of LsoD are assessed in relation to Lso haplotypes A, B, and C, as well as the facultative strain *Liberibacter crescens*. We identify genes unique to haplotype D as well as putative secreted effectors that may play a role in disease characteristics specific to this haplotype of Lso.

## Introduction

*Candidatus* Liberibacter solanacearum (Lso), a species within the genus of *Ca.* Liberibacter and the family Rhizobiaceae of the class alphaproteobacteria, is the suspected causal agent of several plant diseases in the families Solanacea and Apiaceae on multiple continents (Abad et al., 2009; Munyaneza et al., 2010a; Alfaro-Fernández et al., 2012a,b; Mawassi et al., 2018). Lso, as other *Ca.* Liberibacter species, is restricted intracellularly to the plant phloem sieve elements, where it is delivered to and acquired from by psyllid feeding in a circulative persistent mode (Hansen et al., 2008; Secor et al., 2009; Nissinen et al., 2014; Teresani et al., 2014; Mawassi et al., 2018). Thus far, six haplotypes of Lso (A-E and U) have been identified based on single nucleotide polymorphism (SNP) analysis of the 16S rRNA gene, 16S/23S internal spacer region (ISR) and 50S rplJ and rplL ribosomal protein genes (Nelson et al., 2011, 2013; Teresani et al., 2014; Haapalainen et al., 2018).

LsoA, detected in North America and New Zealand, and LsoB, found in North and Central America, are associated with solanaceous crops, including potato, tomato and capsicum, and are transmitted by the psyllid *Bactericera cockerelli* Sulc (Hansen et al., 2008; Liefting et al., 2008, 2009; Secor et al., 2009). The symptoms of solanaceae infected with Lso were first described for potato crops ruined in the United States and Mexico. When freshly cut, infected tubers exhibit browning around the vascular tissues, which intensifies and turns necrotic black upon chip frying, known as zebra chip (ZC) (Abad et al., 2009). Infected plants may also exhibit chlorosis, curling and purpling of leaves, swollen nodes and aerial tubers (Abad et al., 2009; Secor et al., 2009). Some of these symptoms, in particular swollen nodes and chlorotic leaves, are common to tomato and pepper as well. Lso haplotypes C, D, and E infect crops in the family Apiaceae. The C haplotype is spread by *Trioza apicalis* Förster and has been found in plants showing characteristic yellows symptoms including stunting as well as discolored and curled leaves. The C haplotype was observed in Finland (Munyaneza et al., 2010a,b, 2011), Sweden (Munyaneza et al., 2012a), Norway (Munyaneza et al., 2012b), and northern Germany (Munyaneza et al., 2015). Later studies showed that the leaf curling symptom was caused by the psyllid vector *T. apicalis*, and that leaf discoloration is caused by the bacterial infection (Nissinen et al., 2014). Lso haplotype D was found in infected carrot plants in several Mediterranean and North African countries, including Spain (Alfaro-Fernández et al., 2012a) and the Canary Islands in the Atlantic ocean (Alfaro-Fernández et al., 2012b; Nelson et al., 2013), France (Loiseau et al., 2014), Morocco (Tahzima et al., 2014), Greece (Holeva et al., 2017), and Tunisia (Ben Othmen et al., 2018), and is vectored by *B. trigonica* Hodkinson. Disease symptoms associated with LsoD include extensive shoot proliferation (i.e., witches’ broom), leaf curling and discoloration, and a hairy growth of secondary roots. We have recently reported the presence, abundance, and association of Lso in carrot fields in Israel. Our haplotyping analysis suggested that only a single haplotype is currently present in Israel, and it is most similar to haplotype D (Mawassi et al., 2018). The E haplotype, which is also vectored by *B. trigonica*, has been reported in both carrots and celery in Spain (Teresani et al., 2014), as well as in crops in France (Hajri et al., 2017). It is not clear if *T. apicalis* and *B. trigonica*, which vector LsoC and LsoD/E, respectively, have haplotype preference or if they simply coincide with the geographical location of these haplotypes. Some degree of host and vector specificity has been seen in experiments with the potato psyllid *B. cockerelli* that does not effectively transmit LsoB to carrots (Munyaneza et al., 2016) and with the carrot psyllid *B. trigonica* that does not effectively transmit LsoE to potato (Antolinez et al., 2017).

It is still unclear what molecular events lead to the development of Lso disease symptoms. It is also not clear if, and how, *Ca*. Liberibacter species are perceived by host plants. Nevertheless, the defense response hormone jasmonic acid is responsive to psyllid feeding, and salicylic acid and abscisic acid production have been connected to *Ca*. Liberibacter asiaticus (Las) infection (Rosales and Burns, 2011; Martinelli et al., 2012; Xu et al., 2015; Nehela et al., 2018). The crosstalk between these major signaling pathways indicates a complex host response. The host responses may in fact exacerbate disease, as Las infection can cause the induction of callose deposition, which has been proposed to lead to phloem plugging (Kim et al., 2009; Koh et al., 2012). Changes in carbohydrate partitioning and the aberrant accumulation of starch have also been suggested to be a result of Las infection and the cause of disease symptoms (Etxeberria et al., 2009). While *Ca*. Liberibacter species lack a type III secretion system, Sec-dependent secretion of proteins (so called effectors) has been implicated in host defense suppression and induction of cell death by Las infection (Jain et al., 2015; Pitino et al., 2016). Such effectors may also be responsible for disease symptoms, as is the case for some phytoplasma-induced disease symptoms (Sugio et al., 2011).

The genomes of Lso haplotypes A, B, and C have been sequenced (Lin et al., 2011; Thompson et al., 2015; Wang et al., 2017). These sequenced genomes are relatively small at <1.4 MB, with low GC content (∼35%). They harbor prophage sequences and are highly similar to each other. Differences in these genomes may be a route to understanding the genetic basis of host specificity, haplotype-specific disease symptoms, and the evolution of Lso. Rapid evolutionary pressure due to host adaptation, and the obligate intracellular nature of *Ca*. Liberibacter species (Hartung et al., 2011), have contributed to a reduced genome size and have, as a result, limited the environmental conditions in which these bacteria can multiply. Currently, the only culturable Liberibacter species is *Liberibacter crescens* (Fagen et al., 2014a). Comparative genomics between *L. crescens* and unculturable *Ca*. Liberibacter species have attempted to understand the environmental requirements for *Ca*. Liberibacter growth (Fagen et al., 2014a,b; Lai et al., 2016).

In order to better understand the etiology and biology of Lso diseases, we report here the draft genome sequence of Lso haplotype D from Israel, designated as strain ISR100, and present a comparative analysis to other Lso haplotypes, as well as to *L. crescens*.

## Materials and Methods

### Collection and Maintenance of *B. trigonica* Insects and Lso Haplotype Determination

The psyllids used for DNA purification and sequencing of Lso haplotype D were collected from commercial carrot fields using the sweep net method as described by Mawassi et al. (2018). Psyllids were maintained in a temperature-controlled greenhouse (25°C), inside insect rearing cages (BugDorm, Inc.) containing healthy carrot plants. Carrot plants showing typical yellows symptoms were collected from commercial fields and were verified by the method of Nelson et al. (2013) to be colonized by the same Lso haplotype D variant described in Mawassi et al. (2018) and not by phytoplasma or spiroplasma. These plants were used as source plants for psyllid feeding and LsoD acquisition. The acquisition of LsoD by the psyllids was validated as described (Mawassi et al., 2018).

### Genome Sequencing and Assembly

Groups of four psyllid adults or six nymphs reared as mentioned above were collected from rearing cages and immediately processed for genomic DNA (gDNA) isolation. Three DNA extractions were prepared. Two samples containing 6 nymphs each were ground in an osmotically supplemented buffer, and the extracts were clarified by several rounds of centrifugations. The final pellet was resuspended in a Tris-Sucrose buffer (20 mM Tris, pH 8.0–10% sucrose) as described by Neimark and Kirkpatrick (1993). gDNA was then purified from the pellets according to a standard DNA purification method for bacteria (Andreou, 2013). Nymph gDNA was then amplified with Qiagen-REPLI-g Mini yielding the gDNAa sample. A clean-up step (QIAamp DNA Mini Kit) was applied to one of the two gDNAa samples to produce the gDNAac sample. For the third sample, genomic DNA from 4 adult psyllids was prepared according to the CTAB method and labeled gDNActab (Cenis et al., 1993). Final DNA concentrations of each sample are given in Supplementary Table S1. Before being sent for sequencing, all three samples were analyzed by the SNP method (Nelson et al., 2013) and were confirmed to contain DNA of the same Lso haplotype D variant previously identified in Israel, which deviates from haplotype D by one SNP at the 16S rDNA sequence (Mawassi et al., 2018).

Whole genome sequencing was performed on libraries generated from 100 ng of each of the gDNA samples described above (gDNAac, gDNAa, and gDNActab) with the TruSeq DNA nano sample prep kit (Illumina), and the Illumina MiSeq (150 bp, paired-end) at the Technion Genome Center, Haifa. The sequences were quality trimmed using Trimmomatic v0.32 (Bolger et al., 2014). The three libraries were then assembled together using A5-MiSeq assembler (Coil et al., 2015). The CAR tool (Lu et al., 2014) was used to order the obtained contigs using the Lso ZC1 strain as a reference. The Tablet viewer was used to manually inspect the contig assemblies and was also used for determining sequencing depth (Milne et al., 2013).

Differently from the other sequenced Lso genomes, in this assembly, only a single rRNA operon was identified. To resolve the other rRNA operons, the gDNActab (adult psyllids) library was mapped back to the obtained assembly with Bowtie2 (Langmead and Salzberg, 2012). Tablet was used to screen for potential miss-assemblies in the contig containing the rRNA operon. Reads mapping to the rRNA operon and the problematic adjacent regions were extracted and re-assembled with MIRA on EST mode (Chevreux et al., 1999). Three different contigs containing full rRNA operons were recovered and added to the original assembly. The gDNActab library was mapped back against the assembly with the three rRNA operons using MIRA in mapping mode and loaded in gap5 (Bonfield and Whitwham, 2010). Finally, gap5 was used to screen for possible joints between the rRNA operons and the rest of the contigs. This Whole Genome Shotgun project has been deposited at DDBJ/ENA/GenBank under the accession PKRU00000000. The version described in this paper is version PKRU02000000.

### Genome Annotation and Comparison

Genomes used here for comparison to LsoD strain ISR100 were collected from the National Center for Bioinformatics described in detail in Table 1, and submitted to The Joint Genomic Institute (JGI)’s IMG/M (Markowitz et al., 2014) for annotation. Following annotation retrieval from JGI, possible pseudogenes were detected using GenePRIMP (Pati et al., 2010), manually inspected and removed from the EC list (Supplementary Table S2).

**Table 1:** Genome list of obligate and facultative strains of *Candidatus* Liberibacter.

Strain	Main plant Host	Vector	Resource {assembly number/IMG genome ID}	Number of ECs^∗^
LsoA (NZ1)	Tomato and pepper	*Bactericera cockerelli*	NCBI {GCA_000968085.1}	357
LsoB (ZC1)	Potato	*B. cockerelli*	NCBI {GCA_000183665.1}	356
LsoC (Fin114)	Carrot	*Trioza apicalis*	NCBI {GCA_001983675.1}	355
LsoD (ISR100)	Carrot	*Bactericera trigonica*	NCBI (GCA_002918245.2)	358
*Liberibacter crescens* BT-1	Papaya	Unknown	NCBI {GCA_000325745.1}	437


*EC, enzyme commission.*

*^∗^Following annotation, filtering and manual curation.*

In order to ensure that the enzyme set for each species is as complete and valid as possible, we have listed all the unique enzymes annotated by different pipelines and checked the domains of the enzymes. The list is composed of SWISS-PROT proteins (Boeckmann et al., 2003) (release 41.0) assigned with an EC number. All proteins in a species matching an enzyme from the query list with more than 30–40% identity over 80% or more query coverage were considered reliable (Freilich et al., 2005). The final number of ECs annotated for each genome is indicated in Table 1. A list of the unique and different ECs for all Lso haplotypes inspected, as well as for *L. crescens*, is given in Supplementary Figure S1.

Phage identification was carried out using PHASTER (PHAge Search Tool Enhanced Release) (Arndt et al., 2016). COG (Cluster of Orthologous Groups of proteins) classifications of Lso haplotype proteomes were carried out using the WebMGA server (Wu et al., 2011). The average nucleotide identity (ANI) of Lso genomes was calculated using the OrthoANI algorithm (Yoon et al., 2017). Proteomes of Lso haplotypes were analyzed by OrthoFinder in order to identify orthologs and proteins unique to LsoD (Emms and Kelly, 2015). Orthologous group clusters were represented as a Venn diagram.

To reconstruct the phylogenetic relationships between the different Lso haplotypes, the Lso genomes presented in Table 1 plus the rest of the sequenced Lso genomes (FIN111, RSTM, HenneA, R1) were used. The harvest suite was used to extract all putative SNPs between Lso haplotypes (Treangen et al., 2014), obtaining a final alignment with 27,685 columns. IQ-TREE v1.5.5 was used to select the best substitution model (TVM with empirical frequencies) and to compute the maximum likelihood (ML) tree with 1000 ultrafast bootstraps and 5000 SH-aLRT support values (Nguyen et al., 2015; Kalyaanamoorthy et al., 2017). Large Collinear Blocks (bigger than 1 kb), or synteny, between haplotypes were assessed with MAUVE aligner (Darling et al., 2010), with the ZC1 genome as reference, and combined with the ML tree using genoPlotR (Guy et al., 2011).

### Identification of Secreted Proteins

The complete set of LsoD proteins was submitted to the SignalP server http://www.cbs.dtu.dk/services/SignalP/ for Gram-negative bacteria with a D-cutoff value of 0.42, a non-stringent setting for SignalP (Bendtsen et al., 2004). Proteins containing a transmembrane domain were removed. BLAST analysis against all sequenced Lso strains was used to identify secreted proteins unique to LsoD.

### Metabolic Activity Simulations

Metabolic activity simulations were carried out using the expansion algorithm (Ebenhöh et al., 2004), which enables predicting the active metabolic network (expanded) given a pre-defined set of substrates and reactions (Opatovsky et al., 2018). Briefly, the algorithm starts with a set of one or more biochemical compounds acting as source metabolites for a feasible reaction, i.e., a reaction for which all required substrates are available. This reaction is selected out of the reaction pool and added to the network. In an iterative process, the products of the chosen reaction are turned into the new substrates, and so on. Processing of the starting-point compounds by relevant reactions increases the number of available compounds that can act as substrates for other previously inactivated reactions. The network stops expanding when there are no more feasible reactions. The metabolic activity of haplotypes was simulated in the approximated media using implementation of the expansion algorithm as described in Ofaim et al. (2017). Graphical representations were created using R (R Core Team, 2012).

An approximation of the relevant metabolic environment of LsoD was retrieved using the NetSeed algorithm (Carr and Borenstein, 2012) through its implantation in NetCmpt (Kreimer et al., 2012). Based on network topology, the algorithm provided a list of metabolites that were predicted to be externally consumed from the environment, termed here as source metabolites. Computational approximation was required since BM7 media that supports growth of *L. crescens* is not defined.

## Results

### Genomic Sequencing and Annotation of LsoD

**FIGURE 1:**
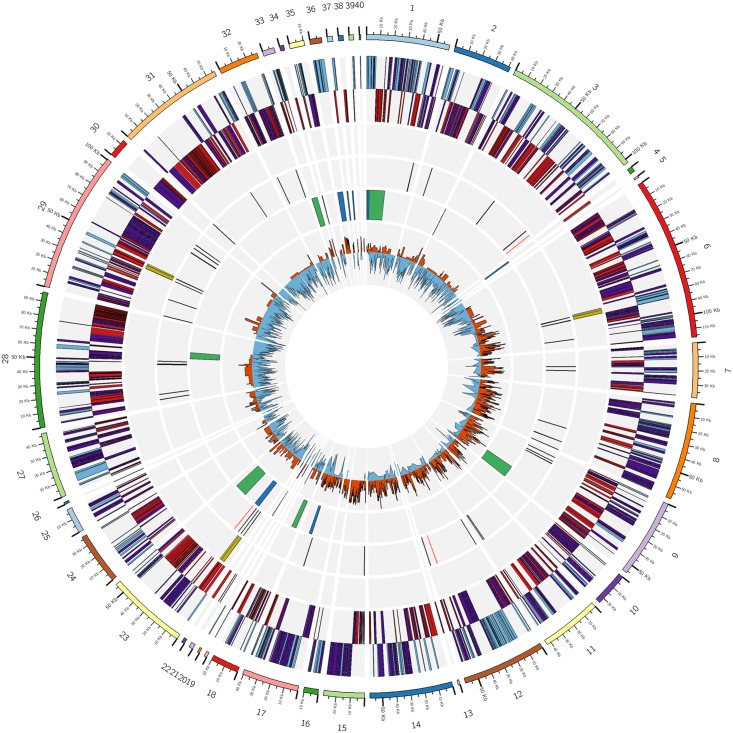
Circular diagram of LsoD ISR100 genome. Descriptions of rings are from the outer to inner circle. (i) Contigs; (ii) protein coding genes in the positive (blue) and negative (red) strands. Enzymes in both strands are highlighted in purple; (iii) rRNA genes (yellow); (iv) tRNA (black lines) and other non-coding RNA (red lines); (v) prophage regions identified by PHASTER (green) and homologous (BLASTN) regions to Prophage P1 from LsoA (blue); and (vi) positive (orange) and negative (blue) GC-skew. Circular ideogram was plotted with CIRCOS (Krzywinski, 2009).

Liberibacter solanacearum haplotype D was chosen to be sequenced from its psyllid host bearing in mind that the higher relative concentration of Lso in psyllids, compared to its carrot host, would aid in the sequencing of the genome (Duan et al., 2009). The low abundance of Lso genomic DNA relative to that of its host led to the use of gDNA amplification with the Q-REPLI-g Mini kit before sequencing in several past genomic studies (Thompson et al., 2015; Zheng et al., 2015; Wang et al., 2017). We attempted to amplify gDNA samples with this kit and also to test if a clean-up step with the QIAamp DNA Mini Kit would lead to better gDNA samples for sequencing. Sequences from the three gDNA-based libraries described above that mapped to Lso were pooled and assembled into 40 non-redundant contigs. Total read number and reads that mapped to our assembled LsoD contigs were compared for these samples (Supplementary Table S1). Total read numbers for amplified nymph DNA were ∼9.6 million reads, in which 0.74% mapped to Lso without a clean-up step and ∼11.4 million total reads with the clean-up step, in which 0.84% mapped to Lso. The unamplified adult psyllid gDNA extracted by CTAB resulted in ∼9.1 million reads, where 3.18% mapped to Lso sequences, approximately fourfold more than the amplified nymph gDNA samples. The LsoD genome’s annotated elements are represented in a circular plot in Figure 1. The average sequence coverage of the assembly is ∼56.2 reads/base. The contigs that assemble into the genome ranged from 1056 bp, containing 3 genes, to 118,316 bp, containing 96 genes. The LsoD genome is 1.30 Mb in size, with 34.8% GC content, and contains 1174 genes, 1128 of which are protein coding (Figure 1 and Table 2). This Whole Genome Shotgun project has been deposited at DDBJ/ENA/GenBank under the accession PKRU00000000. The version described in this paper is version PKRU02000000.

**Table 2:** Features of *Candidatus* Liberibacter solanacearum genomes.

Name	LsoD (ISR100)	LsoA (NZ1)	LsoB (ZC1)	LsoC (FIN 114)
Size (bp)	1,302,651	1,312,416	1,258,278	1,245,124
# of Contigs	40	5	1	5
GC%	34.80%	35.32	35.24%	35.16%
rRNA operons	3	3	3	3
rRNA (5s, 16s, and 23s)	9	9	9	9
tRNA	45	45	45	45
Number of predicted genes	1172	1217	1246	1167
Protein coding genes	1126	1159	1192	1110
With function prediction	860	877	831	851


In our genome assembly, three copies of rRNA operons, containing the 5s, 16s, and 23s rRNAs were identified, and a total of 45 tRNAs. These RNA features are similar to those in genomes of previously sequenced Lso haplotypes (Lin et al., 2011; Thompson et al., 2015; Wang et al., 2017; Table 2).

Six incomplete prophage regions were detected in the genome sequence of LsoD. The largest to the smallest regions are a 24.1 kb region on contig 1, a 16.3 kb on contig 23, a 15.5 kb on contig 9, an 8.1 kb on contig 28, a 7.4 kb on contig 33, and a 5.9 kb on contig 18 (Figure 1). Of these incomplete prophage regions only the one found on contig 23, which is composed of 24 proteins and has a GC content of 33.93%, has homology to the conserved prophage NZ1 P1 (Thompson et al., 2015), ZC1 P1 and P2 (Lin et al., 2011), and FIN114 A (Wang et al., 2017), found in all sequenced Lso. A BLASTN search of the LsoD genome found additional fragments of this conserved prophage in a total of 10 LsoD contigs ranging from ∼178 bp to 6.7 kb (Figure 1 and Supplementary Table S3). The largest incomplete prophage region, consisting of 12 proteins with a GC content of 36.49%, detected on contig 1, did not have homology to previously reported Lso prophage sequences.

### Comparison of LsoD to Lso Haplotypes A, B, and C

**FIGURE 2:**
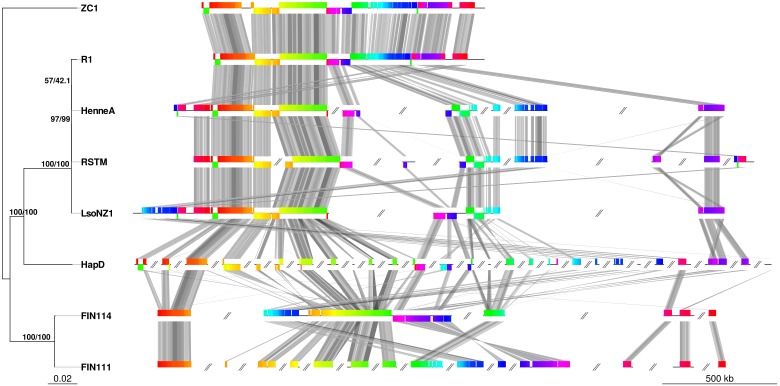
Unrooted maximum likelihood phylogenetic tree based on SNPs shared between Lso haplotypes (left) and synteny blocks common to all the haplotypes (right). Bootstrap (left) and SH-aLRT (right) support values are plotted at each node. Colors of syntenic blocks are in accordance with their position in the reference genome (ZC1). Colors are maintained across genomes and are connected by gray lines. Gray double slash denotes contigs. R1 was plotted as a concatenated set of contigs. Only contigs with common blocks are plotted in the other genomes.

The similarity between Lso genomes was determined using ANI, a common measure used to demarcate microbial species boundaries (Yoon et al., 2017). The highest ANI score was measured between Lso haplotypes D to C, and D to A (ANI of 97.86 and 97.85%, respectively) followed by D to B (97.35%). The greatest divergence was recorded between Lso haplotypes C and A, with an ANI score of 97.18%. As 95–96% is the ANI threshold for species boundaries (Yoon et al., 2017), our results support the current designation of all Lso as haplotypes of the same species (Supplementary Table S4). The phylogenetic tree, obtained from 27,162 SNPs, presented a topology that generally supports the ANI results, being that LsoD is closer to LsoA and LsoC than to LsoB (Figure 2 and Supplementary Figure S2). The draft status of the LsoD genome makes macrosyntenic comparisons with the other haplotypes difficult. However, as was previously shown (Wang et al., 2017), microsynteny among the different haplotypes is generally maintained, as can be seen by the lines connecting conservedas blocks between the haplotypes (Figure 2).

**FIGURE 3:**
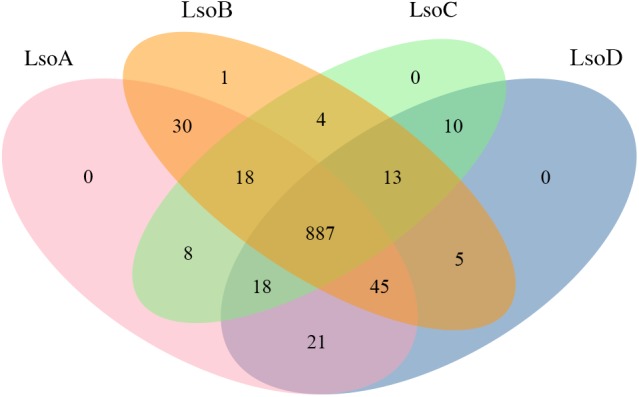
Orthologous clustering of Lso haplotypes determined using OrthoFinder are represented as a Venn diagram. The values indicate the number of orthologous protein families.

The similarity of these genomes led us to investigate the gene content of the different haplotypes. We found a set of 887 orthologous clusters that were shared among all four haplotypes studied here (Figure 3). A large majority of these orthologous clusters, 856 (∼73%), represent single copy genes, indicating that the single copy status of these genes was maintained after the divergence of these haplotypes. This is consistent with the pressure to maintain a small genome, or shrink further, on intracellular pathogens that maintain obligate associations with eukaryotic hosts (McCutcheon and Moran, 2011). Collectively, the four haplotypes formed 1055 orthologous groups. Direct comparison of orthologous clusters found that LsoA and LsoB have more unique common orthologous clusters (30) than LsoA has with LsoD (21). Based on this clustering analysis LsoD has the least unique common orthologs with LsoB (5). LsoD shares 10 unique common orthologous clusters with LsoC. Upon closer inspection by BLAST search, these were all found to be hypothetical proteins with no known function.

We investigated if any protein coding genes of LsoD compared to haplotypes A, B, and C may be unique. This resulted in the identification of 33 genes that are unique to LsoD (Supplementary Table S5) with the BLASTP bit-score cut off at >50. LsoA, B, and C were found to have 102, 69, and 14 unique genes, respectively. The majority of the genes unique to LsoD encode hypothetical small proteins of less than 100 aa, with the exception of seven that are greater than 100 aa. Five have identified putative functions; two may encode a transposase, two are potential restriction endonucleases, and one is putative protease. This is similar to what was reported for LsoC where most of the genes were annotated as hypothetical (Wang et al., 2017).

### Identification of Putative Secreted Factors

Because intracellular bacteria, like phytoplasma, have been shown to use secreted proteins to manipulate host physiology and defense responses, we screened for putative active secretion pathways (Sugio et al., 2011). The Sec pathway components SecA, SecB, SecE, SecY, and SecD are conserved in all Lso haplotypes sequenced and are present in LsoD as well, indicating that the Sec pathway is likely functional (Mori and Ito, 2001). The Sec pathway translocates proteins containing specific N-terminal signal peptides from the cytosol to the periplasm after removal of the signal peptide. While it is not clear how such secreted proteins are then moved into the extracellular space and media, they could be translocated via outer membrane vesicles, autotransporters, or ß-barrel proteins (Costa et al., 2015; Katsir and Bahar, 2017).

We predicted putative secreted proteins (PSPs) containing a N-terminal signal peptide and cleavage sites using the online server SignalP. After removing proteins that contain transmembrane domains, 44 PSPs, which we have designated as LsoD PSPs, were identified. Of the 44 PSPs, 30 proteins had no known function, while those with an assigned function have roles associated with the outer membrane, for example a flagellar P-ring and motor proteins, iron binding proteins, a peptidase, a hydrolase, and a restriction endonuclease. Our analysis identified two PSPs of unknown function that are unique to LsoD (Table 3, marked with asterisk) among the sequenced haplotypes of Lso.

**Table 3:** Predicted LsoD PSPs.

PSP Name	LsoD Gene	IP	Length (aa)	MW (Da)	*D* Score	Cleavage Score	Cleavage Position	Annotation	
PSP1	1014	9.74	304	34789	0.517	0.214	36	M23 family peptidase	
PSP2	1051	7.07	106	11659	0.843	0.82	23	Hypothetical protein	
PSP3	1105	9.29	835	96544	0.443	0.127	11	Restriction endonuclease	
PSP4	1115	5.92	34	3648	0.451	0.305	25	Hypothetical protein	
PSP5	1161	6.16	87	9069	0.496	0.153	25	Hypothetical protein	
PSP6	1187	9.08	111	12621	0.505	0.211	23	Hypothetical protein	**^∗^**
PSP7	1248	4.33	71	7459	0.713	0.426	20	Hypothetical protein	
PSP8	1251	6.27	111	12826	0.612	0.363	21	Hypothetical protein	
PSP9	1274	9	278	31147	0.871	0.721	25	Lytic transglycosylase	
PSP10	1311	7.76	83	9616	0.511	0.221	21	Hypothetical protein	
PSP11	1323	7.74	182	21083	0.455	0.131	11	Hypothetical protein	
PSP12	1373	6.5	282	31901	0.663	0.577	25	Hypothetical protein	
PSP13	1388	7.7	162	18306	0.55	0.57	28	Hydrolase	
PSP14	1419	7.19	363	40791	0.471	0.205	23	Ribonucleotide synthase	
PSP15	1493	5.98	47	4965	0.724	0.388	20	Hypothetical protein	
PSP16	10220	8.67	161	17994	0.727	0.412	24	Hypothetical protein	
PSP17	10410	8.28	310	34962	0.536	0.305	21	ABC transporter substrate-binding protein	
PSP18	10435	9.57	344	38342	0.534	0.226	25	Hypothetical protein	
PSP19	10615	9.16	369	39171	0.622	0.403	21	Flagellar P-ring protein	
PSP20	10616	9.79	149	16034	0.685	0.289	20	Flagellar P-ring protein	
PSP21	10653	8.26	297	33437	0.598	0.409	29	Zinc ABC transporter	
PSP22	10662	8.95	472	51342	0.79	0.539	25	Pillus assembly CpaC	
PSP23	12342	9.2	230	24553	0.813	0.626	24	Outer membran protein	
PSP24	12352	7.83	60	6416	0.488	0.216	29	Hypothetical protein	
PSP25	13149	5.93	188	19565	0.549	0.242	26	Collagen-like protein	
PSP26	13211	9.02	425	49808	0.877	0.851	21	Translocation protein TolB	
PSP27	13212	6.28	152	17247	0.877	0.851	23	Flagellar motor MotB	
PSP28	13221	8.23	299	33945	0.79	0.642	19	Iron binding protein	
PSP29	13472	4.73	92	9671	0.731	0.563	24	Hypothetical protein	
PSP30	13473	10.01	65	7055	0.619	0.181	23	Hypothetical protein	
PSP31	13474	8	81	8684	0.655	0.183	23	Hypothetical protein	
PSP32	13475	6.71	83	8955	0.445	0.213	25	Hypothetical protein	
PSP33	13637	9.57	100	12071	0.733	0.409	26	Hypothetical protein	
PSP34	13716	4.68	38	4286	0.566	0.202	25	Hypothetical protein	^∗^
PSP35	13824	8.56	200	21884	0.62	0.779	28	Hypothetical protein	
PSP36	13912	9.75	154	17413	0.874	0.88	35	Hypothetical protein	
PSP37	13914	7.69	126	14332	0.542	0.173	27	Hypothetical protein	
PSP38	13922	8.33	258	30053	0.499	0.215	23	BamD	
PSP39	13928	9.54	66	7295	0.598	0.26	19	Hypothetical protein	
PSP40	14118	6.58	154	17820	0.868	0.827	23	Hypothetical protein	
PSP41	14433	9.23	107	12295	0.617	0.24	20	Hypothetical protein	
PSP42	14447	8.33	202	23113	0.578	0.15	31	Hypothetical protein	
PSP43	14448	6.19	70	7355	0.481	0.222	22	Hypothetical protein	
PSP44	14660	8.86	106	11692	0.825	0.794	23	Hypothetical protein	


*LsoD putative secreted proteins (PSPs), LsoD gene names are derived from the JGI annotation, isoelectric point (IP), molecular weight (MW), discrimination score (D), ^∗^unique to LsoD (Blast Score <75).*

### Comparative Analysis of the Functional Genome Capacity of LsoD to *L. crescens*

**FIGURE 4:**
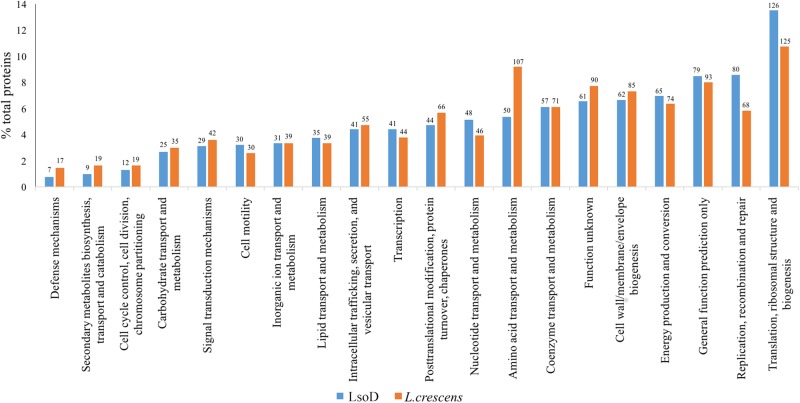
Comparison of clusters of orthologous groups (COGs) assignments determined by the WebMGA server of LsoD and *L. crescens* are plotted as a percentage of their total respective genomes. The absolute number of genes is indicated above each bar.

The functional capacities of LsoD, as inferred from classification of proteins into the clusters of orthologous groups (COGs) scheme, were compared to those of the related *L. crescens* strain (Figure 4). While Lso haplotypes are currently considered uncultivable, *L. crescens*, which possess a slightly larger genome (1.5 MB), has a broader metabolic capacity and can be grown in artificial media (Fagen et al., 2014b; Lai et al., 2016). As expected, *L. crescens* contributes more proteins to most functional groups than LsoD. Most notable are ‘amino acid transport and metabolism,’ ‘defense mechanisms’ and ‘secondary metabolites biosynthesis, transport and catabolism,’ to which *L. crescens* contributes more than two times the proteins than LsoD (Figure 4). On the other hand, LsoD contributes more proteins than *L. crescens* to ‘nucleotide transport and metabolism’ (48 vs. 46 genes) and to ‘DNA replication, recombination and repair’ (80 vs. 68 genes). In the categories for gene ‘motility’ and ‘translation, ribosomal structure and biogenesis,’ LsoD dedicates a slightly larger percentage of genes than *L. crescens*, though the absolute number of genes is similar.

### Evaluation of Metabolic Potential of LsoD

In order to better understand host specificity of Lso haplotypes and their environmental requirements, we compared the set of enzymes (ECs, enzyme commission) of LsoD with LsoA, LsoB, and LsoC, as well as with the culturable haplotype *L. crescens* (Fagen et al., 2014a). LsoD has 358 ECs, similar to the other non-culturable haplotypes (Table 1), with a core group of 352 enzyme accessions. LsoD encoded a single unique EC and shares single unique ECs with A and B but not with C (Supplementary Figure S3). These differences, however, do not manifest in different metabolite requirements between haplotypes. Unique ECs are mapped to pathways involved in cysteine and methionine biosynthesis, terpenoid biosynthesis, and lipopolysaccharide biosynthesis (Supplementary Figure S1). However, these pathways are robust enough to be unaffected by the small haplotype-specific differences predicted.

A comparison to *L. crescens* reveals that the majority of LsoD enzymes (350) are included in the larger set of *L. crescens* enzymes (437) (Supplementary Figure S1). *L. crescens* enzymes absent in LsoD are likely to be responsible for the broader metabolic potential of *L. crescens*, allowing growth in culture. Eight enzymes were found to be present in LsoD but not in *L. crescens*, however, these ECs were found to impart no novel metabolic potential.

Given a representation of data as a network, computational simulations allow for addressing the influence of environmental inputs (nutritional resources) on its structure and composition, i.e., the metabolic capacities of a species in a given environment, for example, in terms of its ability to produce essential metabolites (Opatovsky et al., 2018). More specifically, expansion algorithms generate the set of all possible metabolites that can be produced given a set of starting compounds (source-metabolites) and a set of feasible reactions. We defined the starting compounds as a compilation of nutrients provided by the host psyllid in the environment of *L. crescens*. Our predicted environment was composed of 444 compounds. For each of the Lso haplotypes and *L. crescens* we simulated metabolic activity in the environment and listed a sub-set of essential metabolites predicted to be produced. Our analysis shows that in contrast to *L. crescens*, all Lso haplotypes have lost their ability to produce the electron carrier ubiquinone, glycerol, as well as being unable to produce the L-amino acids alanine, phenylalanine, tyrosine, tryptophan, methionine, histidine, and proline (Supplementary Figure S4).

**FIGURE 5:**
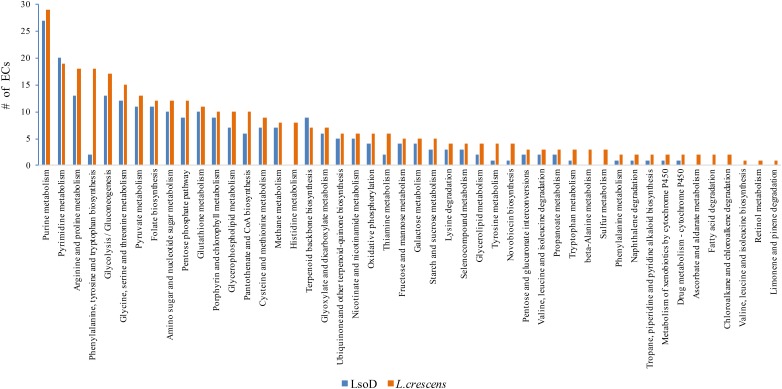
Comparison of the number of ECs for LsoD and *L. crescens* is mapped to metabolic and degradative pathway. The absolute number of ECs for each pathway is graphically represented.

Analysis of the impact of the differences in ECs between LsoD and *L. crescens* on specific pathways reveals entire metabolic and degradative pathways to be missing in LsoD (Figure 5). This includes losses to pathways for the metabolism of retinol, nucleotide sugars, amino sugars, and the amino acids listed above, as well as loss of degradative pathways for fatty acids and terpenes.

## Discussion

Recent assessments of field-grown carrots in Israel have revealed the presence of *Candidatus* Liberibacter solanacearum haplotype D (LsoD) and demonstrated that observed field phenotypes can be replicated in greenhouse conditions (Mawassi et al., 2018). To better understand Lso diseases and the unique and shared characteristics of its haplotypes, the genome of the carrot yellows-associated LsoD from Israel (Mawassi et al., 2018) was sequenced. Genomic DNA prepared by the CTAB protocol from Lso-positive *Bactericera trigonica* psyllid adults, was found, by this study, to yield the highest number of Lso reads. This result was somewhat surprising, considering that whole genome amplification kits are often used to increase the number of reads, hence the coverage, of obligate, intracellular pathogen DNA in mixed samples. Since the DNA for the unamplified and amplified samples originated from psyllids of different developmental stages, adults and nymphs, respectively, we could not conclude whether the higher number of reads was a result of the purification protocol or the developmental stage of the insects. Nevertheless, this result suggests that total DNA extraction from *B. trigonica* adults, using the CTAB method, can be used for whole genome sequencing of Lso without the use of genome amplification kits, which are prone to produce chimeras (Lasken and Stockwell, 2007) that complicate downstream bioinformatics analyses.

The genome of LsoD strain ISR100 is highly similar to the other sequenced haplotypes of Lso, A through C (Table 2). A genomic feature where differences were found among LsoD and the other haplotypes was the prophage sequences. In LsoD, we identified six incomplete prophage regions, including one that is conserved in all sequenced Lso genomes. The other incomplete prophages had no homology to Lso prophage regions reported, however, it is not unusual to find phage-derived or phage-remnant sequences in *Ca*. Liberibacter genomes (Wulff et al., 2014). It is evident from our assembly and BLASTN searches against the conserved *Ca*. Liberibacter prophage that a putative complete prophage is scattered across multiple contigs in the draft genome of LsoD. Additionally, the high number of contigs for this assembly impedes comment on the orientation and arrangement of these genomic features.

Though it can be expected that Lso haplotypes who share similar plant hosts and geographic ranges would be the most similar to each other, this was not the case in this study. First, our ANI results indicated that LsoD is as similar to LsoC as it is to LsoA (less than 0.01% difference). Secondly, our phylogenetic SNP analysis clearly indicated that LsoD is closer to LsoA than it is to LsoC. These results corroborate three previous phylogenetic analyses of Lso haplotypes, based on 16S rRNA, multilocus sequence typing, and on 88 single-copy ortholog groups, which showed that the solanaceous-associated haplotype LsoA was phylogenetically closer to the carrot-infecting haplotypes LsoC and LsoD than to LsoB (Nelson et al., 2013; Wang et al., 2017; Haapalainen et al., 2018). Geographically, Haplotypes D and A are very distant from one another, with D found in the Mediterranean and A found in North America. Haplotypes B and A, on the other hand, not only share the same geographical location, they also share the same vector species, *B. cockerelli* (Nelson et al., 2011), yet they appear to be phylogenetically more distant from one another. These results suggest that haplotype divergence is a complex process, which cannot be explained merely by host association or geographic location. In cases where strains/haplotypes are considered, using quickly evolving genes or SNPs could give a better understanding of the phylogenetic relationships. ANI, alternatively, can give extra information as it considers the entire genome (Konstantinidis et al., 2006), but it should still be considered in combination with other phylogenomic methods.

Our examination of orthologous clusters shared between LsoA, B, C, and D showed 887 orthologous groups shared by the 4 haplotypes examined. In all, 98% of the ortholog groups contain one member from each of the Lso haplotypes. Considering the small genome size of Lso it makes sense that these core conserved proteins are made up of only one member per genome. Inspection of orthologous clusters unique to specific Lso haplotypes or shared between two did not reveal unique functions as the majority of these genes are unidentified. We have also identified 33 genes that are unique to LsoD, however, the majority are small (<100 aa) hypothetical proteins. The proteins identified to be unique to LsoC were reported to be, for the majority, hypothetical proteins as well (Wang et al., 2017). RNASeq- and proteomics-based approaches could be a good starting point to understand the molecular basis of Lso haplotype-specific biology by determining if these proteins are expressed in its different hosts.

Disease symptoms are notably distinct between LsoD and C, though they share similar plant hosts. While LsoD induced the formation of axillary shoot branching, there have been no reports of this phenotype from LsoC-infected carrot plants. Notably, Lso haplotype E has been reported to induce shoot branching in celery (Teresani et al., 2014). We speculate that secreted effector proteins may be involved in the formation of haplotype-specific disease symptoms, including the emergence of axillary branches in LsoD. LsoD PSPs were identified here, as well as resolving that two of these PSPs are unique to LsoD. PSPs have been identified for Lso haplotype C, however, no functional roles have yet been identified (Wang et al., 2017). The majority of the secreted proteins detected are hypothetical proteins, though several enzymes (peptidase, endonuclease, glycosylase, hydrolase, and a ribonucleotide synthase) were detected as well. Further investigations will determine the function of these PSPs.

Our analysis comparing COG functional groups of LsoD to *L. crescens* is a rough overview of the resource allocation of these genomes to particular functional categories. Considering the reduced genome of LsoD, it is expected that less proteins will be contributed to most categorial functions as compared to *L. crescens*. Yet, in some categories, not only did LsoD retain the same number of proteins as *L. crescens* (‘motility,’ ‘translation, ribosomal structure and biogenesis’), it had more proteins allocated to ‘nucleotide transport and metabolism’ and, more significantly, to ‘DNA replication, recombination and repair’ (12 more genes in LsoD than *L. crescens*). It is intriguing to speculate that the larger contribution to replication, recombination, and repair is needed by LsoD to maintain genetic integrity because of its dual host environments, or perhaps because it exists in a population with low genetic diversity (McCutcheon and Moran, 2011). The similar protein content in motility between LsoD and *L. crescens* is interesting, however. Despite having most of the genes required for flagella assembly, flagella were not yet observed in microscopy studies of Lso, and motility via flagella has not been shown in this organism or in *L. crescens*.

Liberibacter solanacearum haplotype D contains less than half the proteins attributed by *L. crescens* to ‘defense mechanisms’ and to ‘secondary metabolites biosynthesis, transport and catabolism.’ This significant difference could be the result of the different environmental exposures of LsoD and *L. crescens*. While LsoD strictly colonizes Apiaceous phloem sieve elements or the psyllid body, *L. crescens* may be exposed to a larger variety of environments including plant surfaces, soil, and insects.

*Liberibacter crescens* is a facultative strain and its greater contribution of genes to ‘amino acid transport and metabolism’ is consistent with its ability to grow in culture. Our metabolic analysis is consistent with this idea as well as finding that Lso, similarly to *Ca.* Liberibacter asiaticus, cannot produce several critical amino acids and cofactors. However, an analysis of metabolic differences between the Lso haplotypes did not reveal differences in the metabolic potential of these genomes determined by identifying their ECs. Further study will be required to determine what the drivers of host specificity are for the Lso haplotypes as well as what pathways Lso exploits to survive in two very different host environments. Understanding the driving factors that determine the relationships between host and vector, whether molecular or geographic in nature, will be important for a complete understanding of this disease.

## Author Contributions

OB conceived the idea. LK, AP, DSG, SF, and OB developed the methodology. LK, RZ, and AP collected the data. LK, RZ, DSG, and NS performed the analysis. LK, RZ, DSG, and OB wrote the manuscript. JJ supervised RZ.

## Conflict of Interest Statement

The authors declare that the research was conducted in the absence of any commercial or financial relationships that could be construed as a potential conflict of interest.
