# Supplementary material for: Genome Analysis of Haplotype D of Candidatus Liberibacter Solanacearum
Source: Front Microbiol. 2018 Dec 10;9:2933. doi: 10.3389/fmicb.2018.02933 (PMC6295461; doi:10.3389/fmicb.2018.02933)
Supplement: Supplementary file 1 [file Presentation_1.pdf]

Supplementary Table S1. Summary of extraction, amplification, and sequencing.

Sample	gDNAac	gDNAa	gDNActab
Source	6 Nymph	6 Nymph	4 Adult Psylid
Extraction method	g-DNA	g-DNA	cTAB
Amplification	Q-REPLI-g Mini*	Q-REPLI-g Mini	No Amplification
DNA ng/µl**	16.9	408	155
Total reads	11,482,272	9,665,080	9,121,560
# of LsoD reads	96,907	71,906	289,941
overall alignment	0.84%	0.74%	3.18%

*amplification was followed by cleanup with QIAamp DNA Mini Kit **final concentration

Supplementary Table S2. Dubious annotation list of *Candidatus* Liberibacter solanacearum haplotype D.

Locus Tag	Dubious annotation
**Ga0157477_1041**	C (+Ga0157477_1201[-119]+Ga0157477_1041[-185]-Ga0157477_1121)
**Ga0157477_1131**	C (+Ga0157477_1231[-239]-Ga0157477_1131[-155]-Ga0157477_1191)
**Ga0157477_1201**	A (+Ga0157477_1031[-368]+Ga0157477_1201[-119]+Ga0157477_1041)
**Ga0157477_1211**	C (+Ga0157477_1081[-173]+Ga0157477_1211[-107]-Ga0157477_1051)
**Ga0157477_1311**	A (-Ga0157477_1051[-317]-Ga0157477_1311[-251]-Ga0157477_1181)
**Ga0157477_1321**	D (-Ga0157477_1191[-1544]+Ga0157477_1321[-184]+Ga0157477_1221)
**Ga0157477_1471**	D (-Ga0157477_1261[-155]+Ga0157477_1471[-107]+Ga0157477_1341)

Supplementary Table S3. Sequences in LsoD with homology to LsoA Prophage P1 identified by BLASTN.

Contig #	% Identity to LsoA Prophage P1	Size (Kb)	Position
3	84	~ 0.2	1-179
1	84	~ 2.7	941-3659
23	98	~ 6.6	11299-17899
17	99	~ 3.4	25044-28450
36	84	~ 6.7	1700-8447
37	80	~ 1.4	1732-3143
4	76	~ 1.8	1627-3394
38	86	~ 1.4	692-2086
34	80	~ 0.6	2236-2825
21	82	~ 0.3	2531-2790

Supplementary Table S4. Percent average nucleotide identity analysis among Lso haplotypes.

	**LsoA (NZ1)**	**LsoB (ZC1)**	**LsoC (FIN114)**	**LsoD (ISR100)**
**LsoA (NZ1)**	100	X	X	X
**LsoB (ZC1)**	97.52	100	X	X
**LsoC (FIN114)**	97.18	97.27	100	X
**LsoD (ISR100)**	97.85	97.35	97.86	100

Supplemental Table S5. List of genes unique to *Candidatus* Liberibacter solanacearum haplotype D.

#	Gene ID	Length (Amino Acids)	Description
1	Ga0157477_10665	62	hypothetical protein
2	Ga0157477_10826	48	putative ISL3 family transposase
3	Ga0157477_1112	93	putative protease
4	Ga0157477_1114	51	hypothetical protein
5	Ga0157477_11610	44	hypothetical protein
6	Ga0157477_11611	121	hypothetical protein
7	Ga0157477_11613	62	restriction endonuclease
8	Ga0157477_1167	45	hypothetical protein
9	Ga0157477_1169	110	hypothetical protein
10	Ga0157477_1184	92	hypothetical protein
11	Ga0157477_1185	102	hypothetical protein
12	Ga0157477_1201	40	hypothetical protein
13	Ga0157477_1202	95	hypothetical protein
14	Ga0157477_1211	36	hypothetical protein
15	Ga0157477_1244	108	hypothetical protein
16	Ga0157477_1285	120	restriction endonculease
17	Ga0157477_1286	48	trasnposase
18	Ga0157477_13139	99	hypothetical protein
19	Ga0157477_13140	65	hypothetical protein
20	Ga0157477_13148	134	hypothetical protein
21	Ga0157477_1323	183	hypothetical protein
22	Ga0157477_13442	30	hypothetical protein
23	Ga0157477_13712	38	hypothetical protein
24	Ga0157477_13716	39	hypothetical protein*
25	Ga0157477_13717	60	hypothetical protein
26	Ga0157477_1384	71	hypothetical protein
27	Ga0157477_13843	75	hypothetical protein
28	Ga0157477_13918	76	hypothetical protein
29	Ga0157477_13929	133	hypothetical protein
30	Ga0157477_1401	50	hypothetical protein
31	Ga0157477_14614	38	hypothetical protein
32	Ga0157477_1471	36	hypothetical protein
33	Ga0157477_1187	112	hypothetical protein*

* putative secreted protein
