# Supplementary material for: Genome Analysis of Haplotype D of Candidatus Liberibacter Solanacearum
Source: Front Microbiol. 2018 Dec 10;9:2933. doi: 10.3389/fmicb.2018.02933 (PMC6295461; doi:10.3389/fmicb.2018.02933)
Supplement: Supplementary file 2 [file Presentation_2.pptx]

## Slide 1
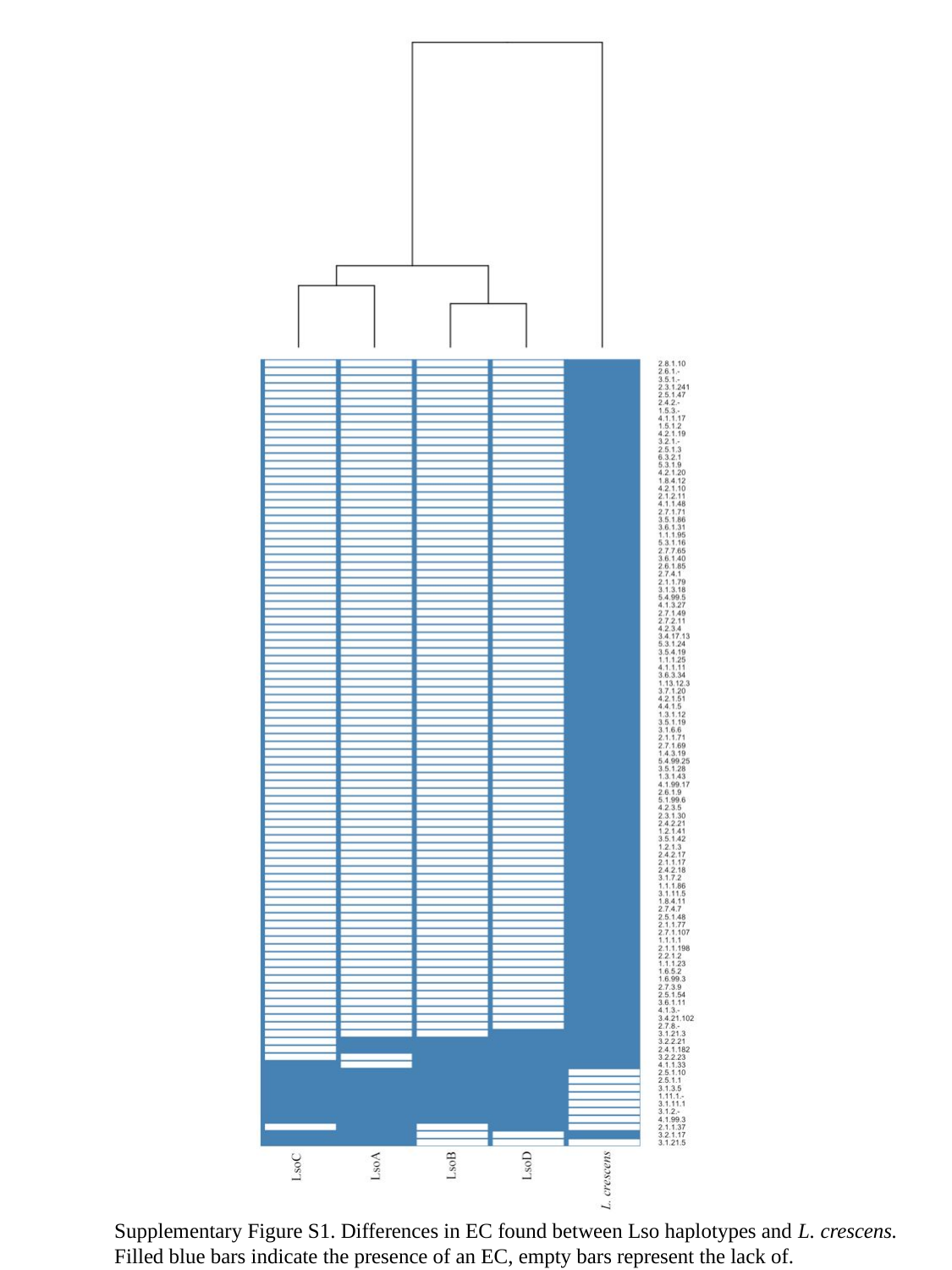

Supplementary Figure S1. Differences in EC found between Lso haplotypes and L. crescens.
Filled blue bars indicate the presence of an EC, empty bars represent the lack of.

## Slide 2
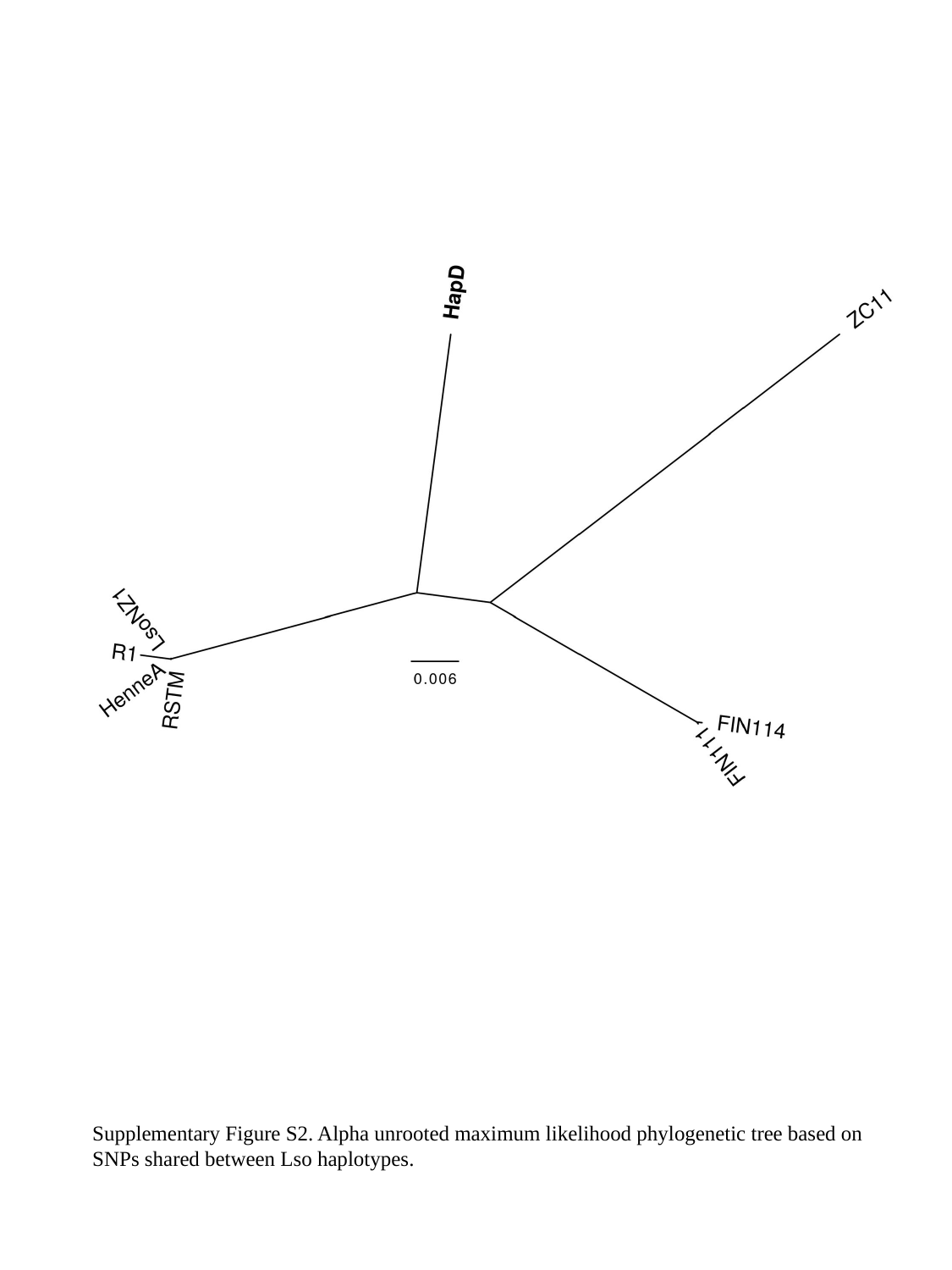

Supplementary Figure S2. Alpha unrooted maximum likelihood phylogenetic tree based on SNPs shared between Lso haplotypes.

## Slide 3
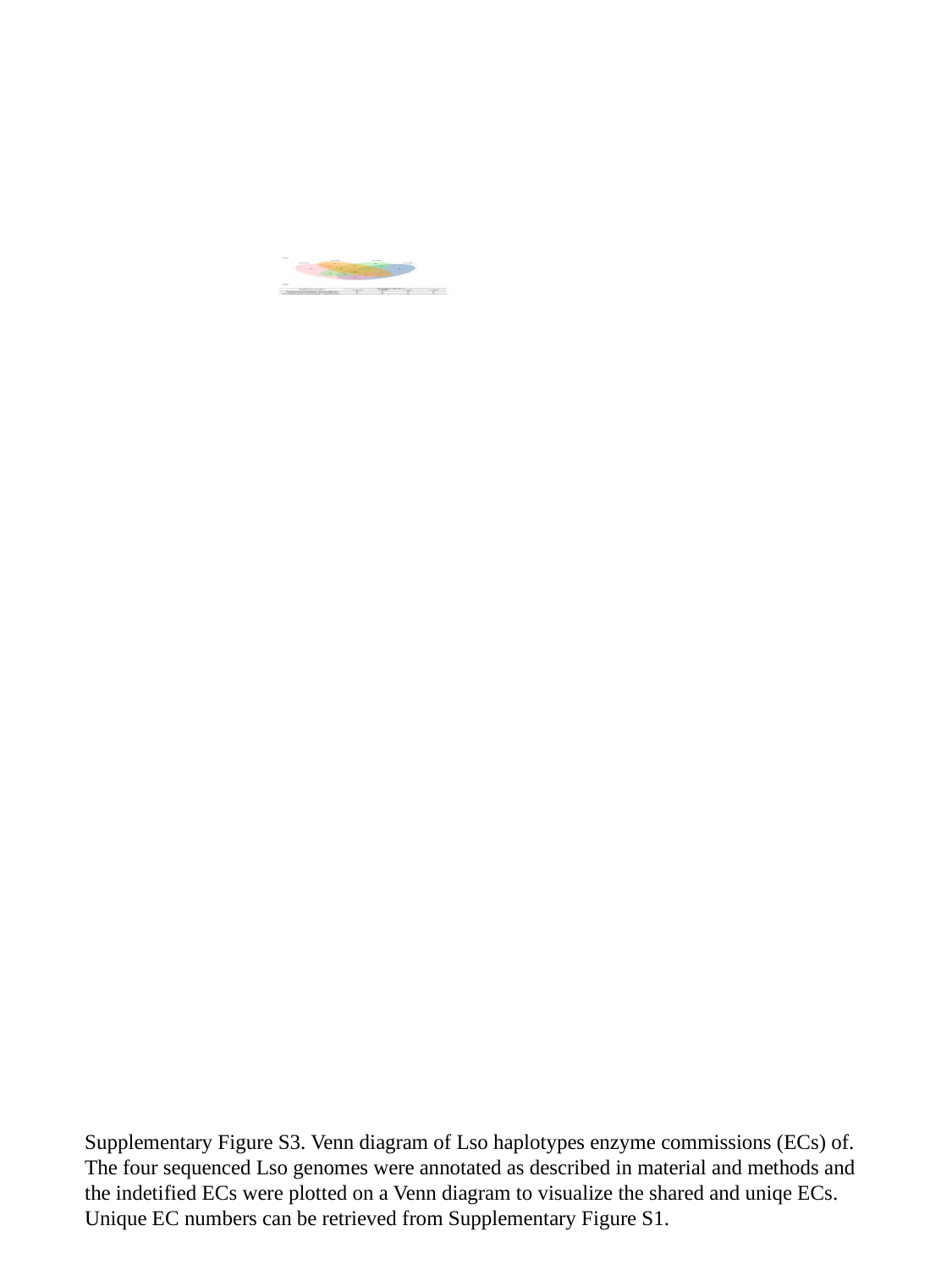

Supplementary Figure S3. Venn diagram of Lso haplotypes enzyme commissions (ECs) of. The four sequenced Lso genomes were annotated as described in material and methods and the indetified ECs were plotted on a Venn diagram to visualize the shared and uniqe ECs. Unique EC numbers can be retrieved from Supplementary Figure S1.

## Slide 4
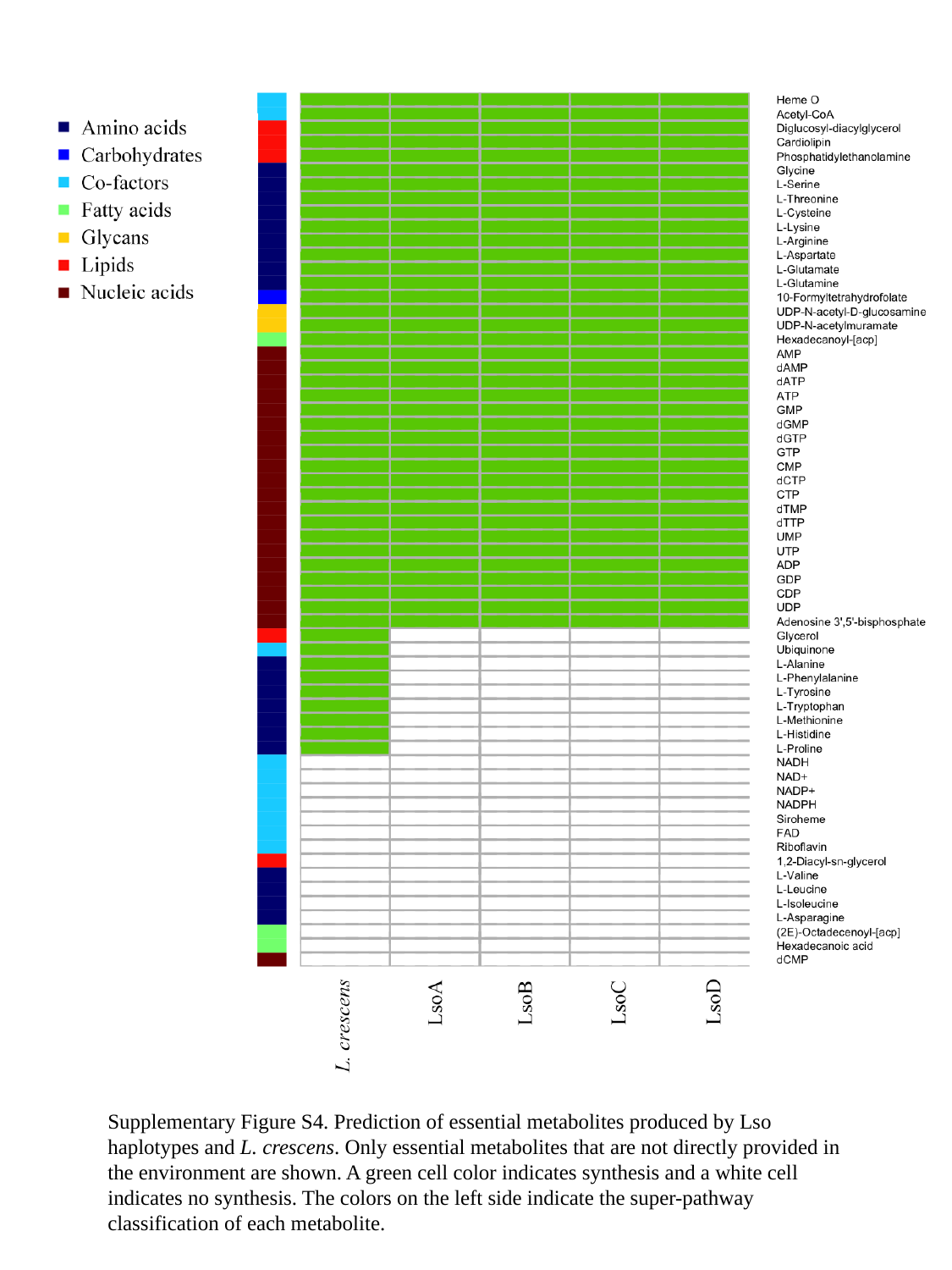

Supplementary Figure S4. Prediction of essential metabolites produced by Lso haplotypes and L. crescens. Only essential metabolites that are not directly provided in the environment are shown. A green cell color indicates synthesis and a white cell indicates no synthesis. The colors on the left side indicate the super-pathway classification of each metabolite.
